# Quantifying Vagus Nerve Stimulation Outcomes in Multifocal Refractory Epilepsy: A Model Across Multiple Surgeries

**DOI:** 10.7759/cureus.69284

**Published:** 2024-09-12

**Authors:** Rida Zakar, John-Victor El Khoury, Gilles Prince, Marc Boutros, Céline Yaghi, Marianne Matar, Karine Abou Khaled, Ronald Moussa

**Affiliations:** 1 Medicine, Université Saint-Joseph, Beirut, LBN; 2 Neurology, Hôtel-Dieu de France, Beirut, LBN; 3 Neurosurgery, Hôtel-Dieu de France, Beirut, LBN

**Keywords:** severity scoring, neuromodulation, clinician-measured outcomes, refractory epilepsy, multifocal epilepsy, vagus nerve stimulation

## Abstract

Objective

This study aims to develop a quantifiable model for evaluating the outcomes of vagus nerve stimulation (VNS) in patients with multifocal refractory epilepsy, particularly focusing on those who have undergone multiple surgeries. By adopting a patient-centered approach, the study seeks to provide a robust framework for assessing VNS efficacy across various patient demographics, including both adult and pediatric patients, and those with impaired cognitive and communicative abilities.

Methods

We conducted a retrospective analysis of 49 patients with multifocal refractory epilepsy who underwent at least one VNS surgery. The cohort was divided into two groups: adults (≥16 years) and a combined pediatric group that included patients under 16 years of age and patients with impaired cognitive and communicative skills. The Liverpool Seizure Severity Scale (LSSS) was used for adults, while the Hague Seizure Severity Scale (HASS) was employed for the pediatric group. Key outcome measures, including changes in seizure frequency, quality of life (QoL), number of hospitalizations, and other clinical metrics, were quantified using our proposed model. The iterative use of the mentioned scales was also assessed for validity by comparison with the Engel Outcome Scale (EOS). A total of 96 procedures were assessed.

Results

The results indicated a significant reduction in seizure severity post-surgery across both groups, as quantified by the LSSS for adults and HASS for pediatric and cognitively impaired patients. The model also demonstrated a consistent decrease in seizure frequency and an improvement in QoL metrics over successive surgeries. Minimal major side effects were reported, supporting the effectiveness of our quantification approach in capturing VNS outcomes.

Conclusions

This study introduces a novel, quantifiable model for evaluating VNS outcomes, providing a comprehensive tool for clinicians to assess the effectiveness of VNS in managing multifocal refractory epilepsy. By integrating multiple outcome measures into a cohesive framework, our model can aid in better understanding VNS therapy’s impact and contribute to more informed clinical practice.

## Introduction

Epilepsy affects approximately 50 million people worldwide, with a prevalence ranging from 0.5% to 1.0%, making it one of the most common neurological disorders globally [1]. Patients with epilepsy have a higher mortality rate and a significantly increased risk of comorbid conditions such as depression compared to the general population [2]. The unemployment rate among people with epilepsy is 33%, which is much higher than the general population [3].

The first line of treatment for epilepsy is usually anti-epileptic drugs (AED), which include carbamazepine, valproate, and lamotrigine, which are anti-seizure medications designed to reduce the frequency and intensity of seizures [4]. However, about 30% of the patients suffer from drug-resistant epilepsy (DRE) [5]. If seizures continue to happen, other treatments like ketogenic regimens, deep brain stimulation, responsive neurostimulation, and epilepsy surgery can help control seizures [6].

In unifocal epilepsy, epilepsy surgeries like the resection of the epileptic focus can contribute to the management. In other cases where the foci are present in one of the hemispheres of the brain, hemispherectomy or callosotomy can be employed. However, in multifocal epilepsy, management by resection surgery can be difficult, and other therapies can be used.

Vagus nerve stimulation (VNS) surgery represents a potential treatment for DRE. VNS is a type of neuromodulation designed to change how brain cells work by giving electrical stimulation to certain areas involved in seizures.

People subjected to VNS showed notably improved chances of achieving a ≥50% decrease in seizure occurrence, a ≥75% decrease in seizure frequency, and a diminished likelihood of requiring higher doses of anti-seizure medications. Contraindications for VNS implantation include post-left-sided vagotomy status, exposure to therapeutic ultrasound, and specific electrotherapies involving current or energy flow through the body.

Quality of life (QoL) is significantly diminished in DRE, with an increased morbidity and a two-to-three-fold elevated risk of mortality. The two most frequently used questionnaires to assess the effect of epilepsy over QoL, while insisting on seizure severity, are the Hague Seizure Severity Scale (HASS or HSSS) and the Liverpool Seizure Severity Scale (LSSS).

The HSSS is a parent-completed questionnaire for measuring the severity of seizures in children with epilepsy. It includes parameters such as frequency of impaired consciousness, duration of impaired consciousness, overall seizure severity, frequency of jerks or cramps, duration of jerks or cramps, noticeability of altered behavior during a seizure, and frequency of confusion during or after an attack.

The LSSS, developed by Baker et al. [7], aims to quantify patients’ subjective perceptions of changes in seizure severity. Initially, the LSSS comprised two subscales: perception of control, consisting of six items, evaluates the overall impact of epilepsy on the patient’s life, considering factors like the timing and predictability of seizures, as well as the presence of an aura. The ictal/post-ictal subscale, consisting of 10 items, summarizes experiences during and immediately following a seizure, including phenomena such as loss of consciousness, post-ictal confusion, headache, and any resulting injury.

A certain nuance exists in regard to QoL and seizure intensity, and the literature surrounding the effects of VNS on intensity specifically is scarce. In this study, effects of VNS on intensity, as well as other parameters, are evaluated. This study is part of a broader pilot study that aims to propose a quantifiable model to evaluate the effects of VNS. Thus, its impact and place in the management of multifocal refractory epilepsy can be better understood and could perhaps contribute to clearer guidelines and indications of VNS therapy.

## Materials and methods

Studied population

The initial target population was comprised of 49 patients who had undergone at least one surgery related to VNS. This could include either implantation of the device only or implantation with at least one revision procedure, a revision procedure being either a change of battery or the management of a device-related complication.

The study included patients with epilepsy classified as "pharmaco-resistant" according to the International League Against Epilepsy [8]. This means that they did not respond adequately to treatment with at least two different antiepileptic drugs for two years. Patients with unifocal epilepsy were excluded, while those with multifocal epilepsy were included unless they were candidates for other surgical procedures like hemispherectomy or callosotomy.

The total population was divided into two groups. Patients considered to be part of the “adult population” were patients aged over 16 years old with intact cognitive and communicative abilities. Patients considered to be part of the “pediatric population” were patients aged under 16 years old or patients who were biologically adults but with impaired cognitive and communicative abilities (unable to respond).

Data collection

The patients were interviewed and offered a questionnaire that assessed parameters before and after each intervention. Additionally, electronic medical records and clinician notes (neurologist and neurosurgeon) were reviewed to supplement the data obtained from the questionnaire.

Both study groups received similar questionnaires comprised of questions related to the patient’s illness and age-adapted scales. The questionnaires were directed either toward the patients themselves or toward their parents.

Adult patients were assigned a questionnaire that included the LSSS 2.0 [9,10] to assess severity. Pediatric patients were assigned a questionnaire that included the HSSS [11,12] to assess severity. All scales were officially translated and communicated to the patients during the interview. Original versions of the scales were offered to the patients who were able to respond in the original language.

To evaluate overall changes in mood and QoL, a model that was similar to the one used in a study by Tractenberg et al. [13] was used. The model focused on changes in QoL and mood, and it used a scale with four answer choices: improved, worsened, remained negative, or remained positive.

To assess changes in seizure frequency, number of hospitalizations, and number of medications before and after the intervention, the data was categorized, and before-and-after scores were analyzed with either Fisher’s exact test or Student’s t-test.

The overall success of the intervention was evaluated by the Engel Outcome Scale (EOS). The score was placed at the end of the interview. It was based on the detailed description of the patient's medical history and their reported results and was then adjusted according to the clinician's notes and the electronic medical records.

The scales were chosen as outcome measures for this study due to their comprehensive ability to capture both seizure severity and overall clinical outcomes. The LSSS 2.0 and HASS are well-established scales that have been validated for measuring seizure severity, making them suitable for quantifying changes pre- and post-VNS surgery. The Engel classification, although traditionally used in resective surgery, was included to provide a broader context of seizure outcomes that may align with reductions in seizure frequency and severity. These measures together allow for a holistic assessment of VNS efficacy, accommodating both qualitative and quantitative aspects of patient outcomes.

Statistical analysis

All analyses were performed on R 4.3.1 (The R Foundation for Statistical Computing, Vienna, Austria) and tabulated on Microsoft Excel. Continuous variables were summarized as median and first and third quartiles. For categorical variables, frequencies and proportions were used. Statistical comparison of continuous variables was performed using Student's t-test. A normal distribution of residuals was accepted after visual verification using the Q-Q graph and if the Jarque-Bera test was >0.05. The F-test was adopted to test for homoscedasticity. Categorical variables and baseline T-scores were compared using Pearson's chi-squared test or Fisher-exact test when n.p or n.(1-p). Type I error estimates were tolerated if they were less than 0.05. Fixed-effects multiple linear regression was performed to attempt to explain LSSS and HSSS scores.

## Results

Population structure and demographics

**Figure 1:**
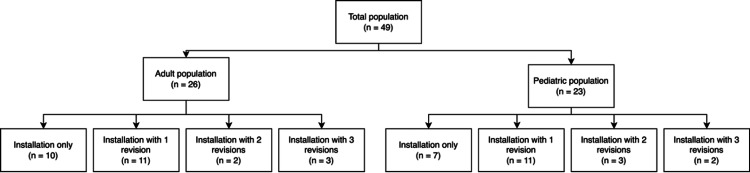
Schematic representation of the distribution of 96 vagus nerve stimulation interventions among adult and pediatric populations The figure illustrates that out of the 96 total surgeries, 49 were performed on adults (≥16 years) and 47 on pediatric patients (<16 years) or those with impaired cognitive and communicative abilities. The figure also shows the number of initial surgeries and subsequent revision surgeries for each group.

Out of 75 patients or parents contacted, a total of 49 responses were obtained. The final sample of the study was therefore composed of 49 patients who had undergone at least one surgery, either a single placement or placement with one or more revisions. A total of 96 surgical procedures were therefore studied. The general population structure can be visualized in Figure 1.

There were 26 individuals belonging to the adult population and 23 individuals belonging to the pediatric or disabled population. There were 29 biological males (59.18%) and 20 biological females (40.82%).

**Figure 2:**
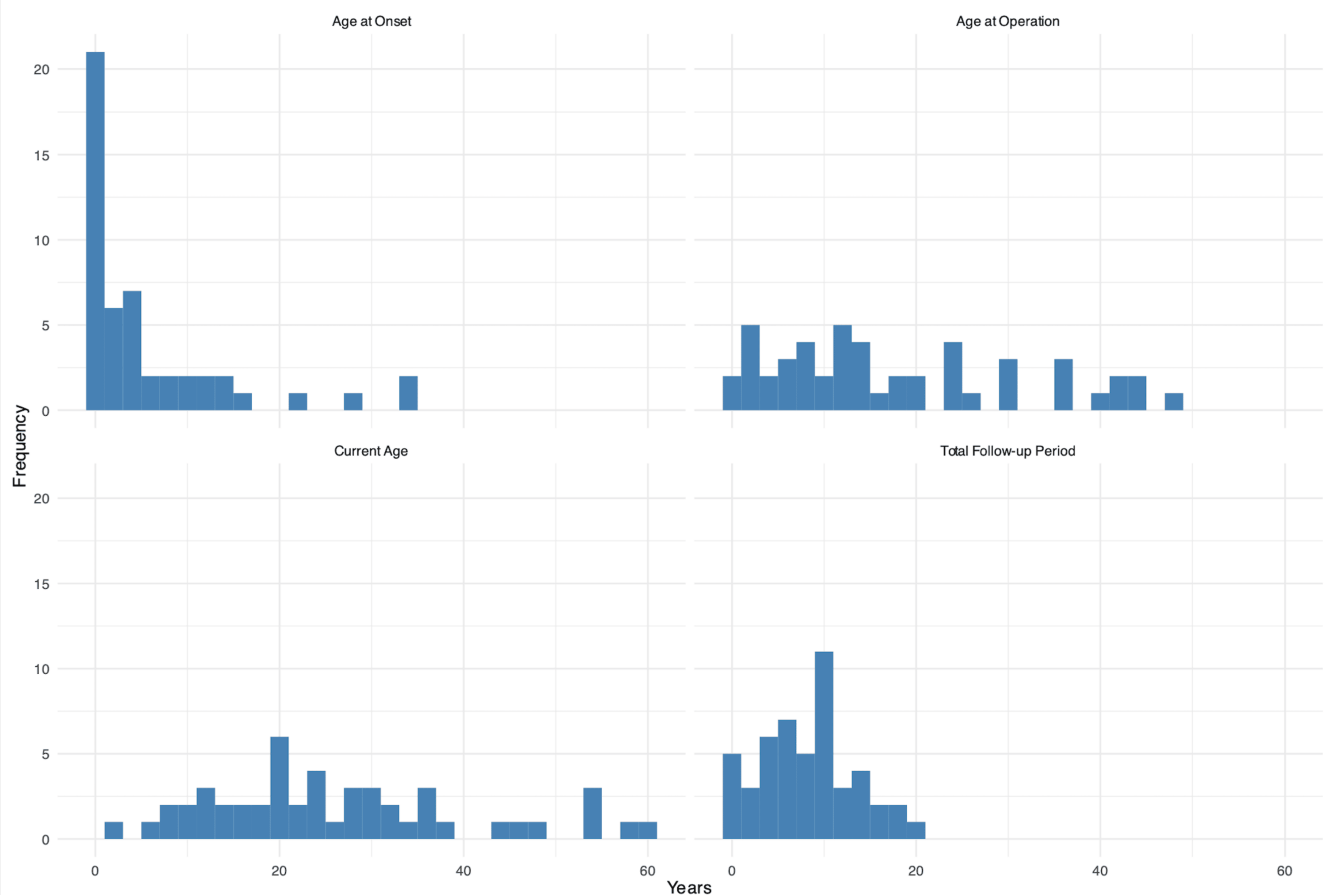
Bar charts summarizing demographic characteristics and patient follow-up details The charts display key variables including age at epilepsy onset, age at surgery, current age, and total follow-up duration. The data reveal that the median age of epilepsy onset was three years, with a median age at surgery of 14 years, indicating early disease manifestation and intervention. The current median age of participants was 25 years, reflecting a wide range of follow-up durations. The total follow-up duration had a median of nine years, providing substantial long-term data on the effects of vagus nerve stimulation.

The median age of epilepsy onset was three years (1st quartile 0.5-3rd quartile 8). The median age at surgery was 14 years (8-27). The median current examination age was 25 years (17-34). The median duration of medical treatment before surgery (implying diagnosis-to-surgery date) was 10 years (4-14). Moreover, the delay of diagnosis can be deduced from the date of seizure onset to beginning of medical treatment (seven years). The median total follow-up duration was nine years (5-11). The demographic data can be summarized in Figure 2.

Of note, most revisions made were battery replacements. One case was documented where the patient had skin thinning above the battery compartment, which subsequently led to the surgical removal of the device. Another case involved the disconnection of electrodes, which was adjusted by revision surgery.

Epilepsy causes

The identified causes of epilepsy were distributed as follows: idiopathic (48.98%), neonatal hypoxia (12.24%), meningitis (10.2%), congenital disease (8.16%), genetic disease (6.12%), neonatal fever or hyperthermia (4.08%), and other causes (hematoma, prematurity, traumatic or tumoral causes) (10.2%).

Side effects

Side effects were reported as major side effects and minor side effects. Very few major side effects like bradycardia, implantation site infections, and electrode disconnection were detected. Of note, although sleep apnea has been documented in the literature as a side effect of VNS [14], not all the patients underwent sleep studies. Thus, this side effect was reported as sleep disturbances rather than diagnosed obstructive sleep apnea.

**Figure 3:**
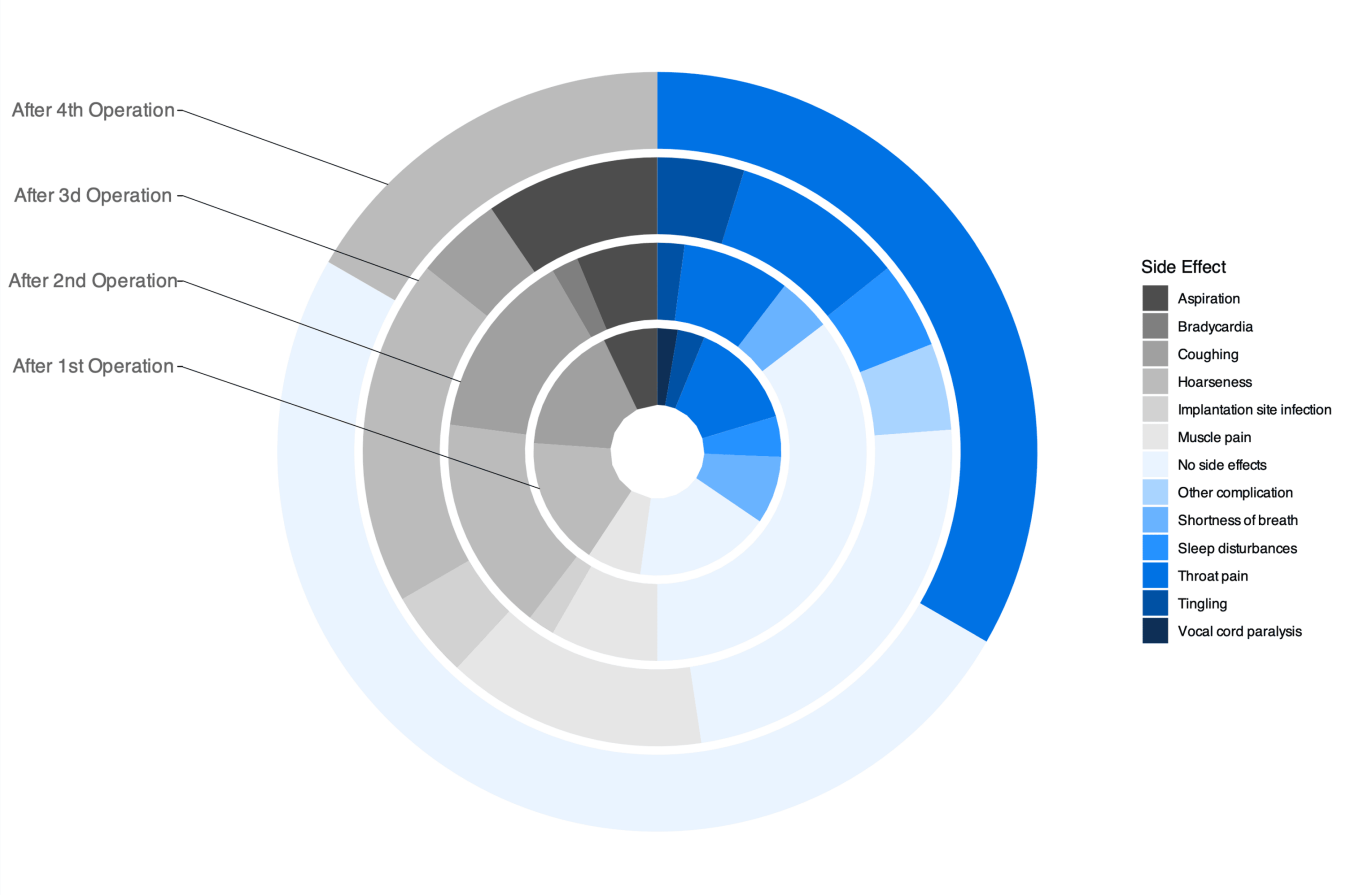
Overlaid pie charts representing the side effect proportions after each surgery This figure illustrates the percentage of patients experiencing various side effects after each surgery. The overlaid pie charts show a trend toward the three main persistent side effects: dysphonia, sore throat, and cough.

The proportion of complications and side effects observed varied from one procedure to another. It was important to note that the most prevalent proportion was that without side effects. Among the other most frequent side effects, dysphonia, sore throat, and cough were noted. Figure 3 gives a visual comparison of the variation in percentages. It is important to note that, after each procedure, the percentage of patients without side effects increased, while dysphonia and sore throat remained. A more detailed outlook on the occurrences can be summarized in Table 1.

**Table 1:** Proportions of side effects following each vagus nerve stimulation surgery The table represents the frequency and percentage of side effects experienced by patients after the first, second, third, and fourth vagus nerve stimulation surgeries. Percentages are calculated based on the total population, not the total number of occurrences. This illustrates the prevalence and trends of specific side effects, such as hoarseness, throat pain, coughing, shortness of breath, and others, across multiple surgeries.

	1st operation	2nd operation	3rd operation	4th operation
Hoarseness	19 (38.78)	8 (16.33)	4 (8.16)	1 (2.04)
Throat pain	16 (32.65)	4 (8.16)	2 (4.08)	2 (4.08)
Coughing	19 (38.78)	7 (14.29)	1 (2.04)	0 (0)
Shortness of breath	10 (20.41)	2 (4.08)	0 (0)	0 (0)
Tingling	4 (8.16)	1 (2.04)	1 (2.04)	0 (0)
Muscle pain	8 (16.33)	4 (8.16)	3 (6.12)	0 (0)
Implantation site infection	0 (0)	1 (2.04)	1 (2.04)	0 (0)
Bradycardia	0 (0)	1 (2.04)	0 (0)	0 (0)
Vocal cord paralysis	3 (6.12)	0 (0)	0 (0)	0 (0)
Aspiration	8 (16.33)	3 (6.12)	2 (4.08)	0 (0)
Sleep disturbances	6 (12.24)	0 (0)	1 (2.04)	0 (0)
Other side effects	0 (0)	0 (0)	1 (2.04)	0 (0)
No side effects	20 (40.82)	17 (34.69)	5 (10.2)	3 (6.12)

In addition, the temporality of these side effects was studied and showed an increased permanence of the noted side effects after the fourth procedure. The variation of temporality can be summarized in Table 2.

**Table 2:** Duration of side effects expressed as percentages of permanent and temporary side effects for each surgical intervention The table displays the proportion of side effects that were permanent or temporary after each vagus nerve stimulation surgery. This data assesses the persistence of adverse effects following vagus nerve stimulation surgeries and identifies any trends in the duration of side effects over multiple interventions.

Side effect duration	1st operation	2nd operation	3rd operation	4th operation
Permanent	17 (56.67%)	7 (50.00%)	4 (66.67%)	2 (100%)
Temporary	13 (43.33%)	7 (50.00%)	2 (33.33%)	0 (0%)

Overall VNS outcomes

Upon interpreting the Engel Outcome Class distribution of the total population, it can be noted that class III (worthwhile improvement) was the most prominent class after all surgeries and revisions, with a percentage of 36.73%. Only approximately the third (26.53%) of the population had class IV negative outcomes. In contrast, the remaining two-thirds can be considered to have a positive outcome.

**Figure 4:**
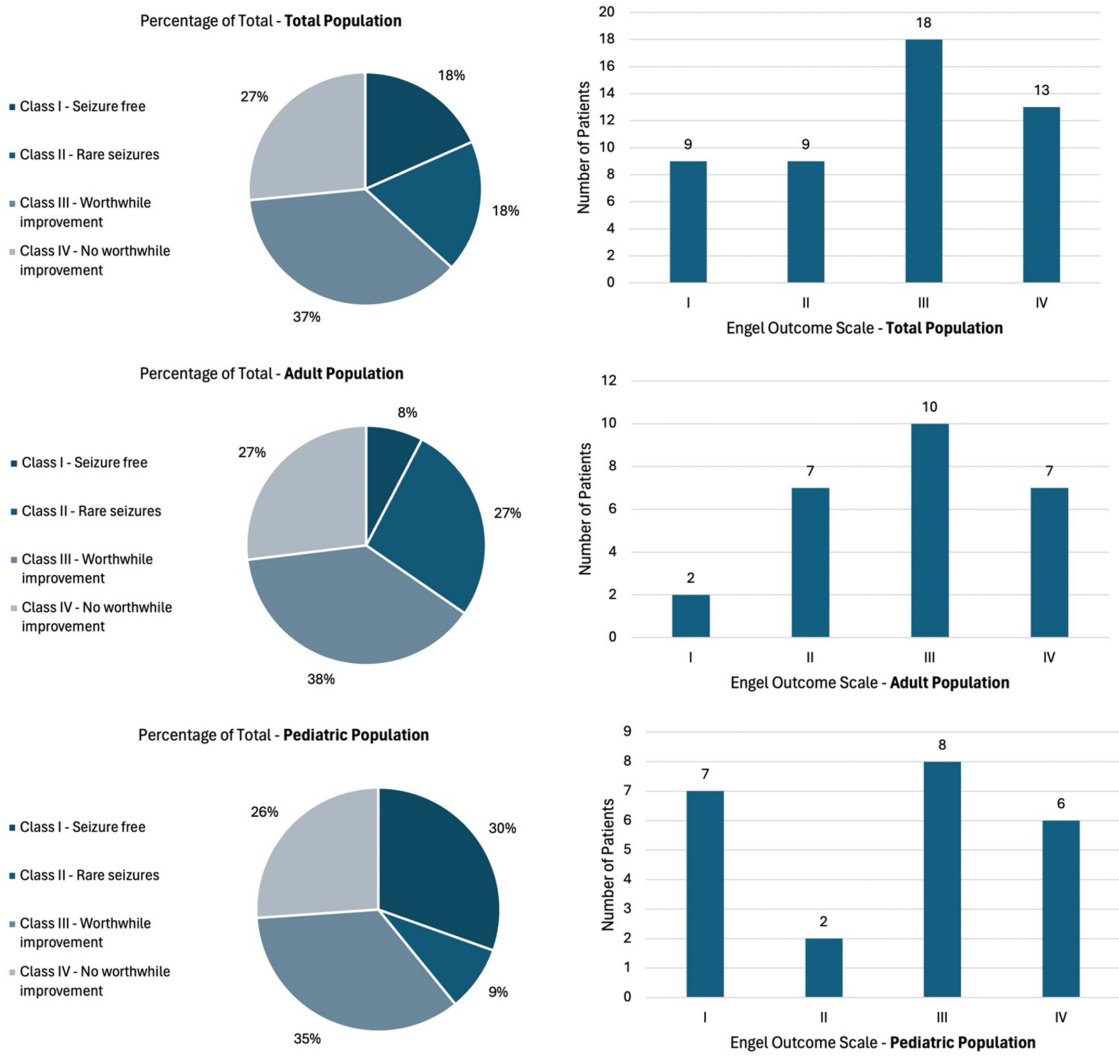
Diagrams illustrating the Engel Outcome Scale distribution in the study populations This figure shows the classification of patients into different Engel Outcome Classes based on their seizure outcomes post-vagus nerve stimulation (VNS) surgery. Of note, class III (worthwhile improvement) was the prominent class (27%), and two-thirds experienced at least a positive outcome.

Class IIIA was the most frequent subscale with a count of 18 over 49. Detailed results can be illustrated in Figure 4. Age-related EOS in concordance with LSSS and HSSS was also analyzed.

Evaluation of changes in seizure severity in the adult population

**Figure 5:**
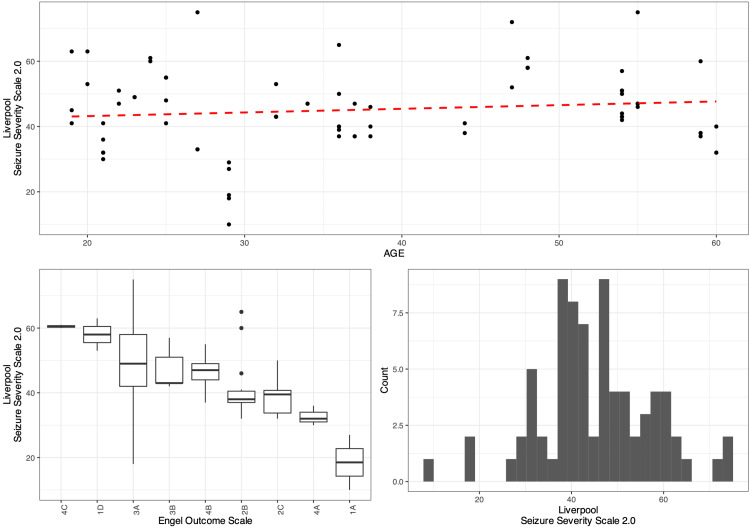
Diagrams illustrating the relationship between Engel Outcome Scale scores and Liverpool Seizure Severity Scale in the adult population The figure shows that patients who achieved better Engel Outcome Scale (EOS) outcomes generally had lower Liverpool Seizure Severity Scale (LSSS) scores, indicating less severe seizures. Specifically, the mixed-effects linear regression model revealed that LSSS scores decreased significantly after each surgery, with an average reduction of approximately 8.38 points after the initial installation (p < 0.001) and further reductions following each revision, highlighting the effectiveness of vagus nerve stimulation (VNS) in reducing seizure severity in the adult population.

The scatter plot in Figure 5 indicates that the LSSS 2.0 increases modestly with age, while the boxplots show that the Engel Scale exposes substantial patient differences in LSSS with respect to their median patient LSSS. Finally, the histogram shows that LSSS 2.0 roughly has a slight negative skew, although grossly normal, with a median of 43.5 (37.5-52.5).

A mixed-effect linear regression model which contained the EOS scores as a random effect was fit to the data in a step-wise-step-up procedure. Due to the presence of outliers in the data, weights were included in the model which led to a significantly improved model fit compared to an un-weight model (χ^2^ (2): 23.80, p = 0.0025). The final minimal adequate model performed significantly better than an intercept-only base-line model (χ^2^ (1): 18.52, p = 0.0024) and showed that the LSSS decreases significantly and substantially after surgery (LSSS0 47.32 vs. LSSS1 47.32 - 8.32 ~ 38.94, and then nearly a point less with each revision) (p < 0.001, marginal R^2^ = 0.145, conditional R^2^ = 0.599). Age had no significant effect on LSSS.

Moreover, a patient's grade on Engel's Scale affects how the LSSS, specifically the standard deviation between a patient's LSSS and another accounting for one's Engel and revision is 3.84 points. A more detailed progression of the model can be visualized in Table 3.

**Table 3:** Mixed effects multiple linear regression model for the adult population using the Liverpool Seizure Severity Scale The table presents the estimated effects of vagus nerve stimulation surgeries and revisions on LSSS scores for adult patients with multifocal refractory epilepsy. The baseline LSSS score (LSSS0) before any intervention was 47.32. Following the initial VNS installation (LSSS1), there was a significant reduction of 8.38 points (p < 0.001). Subsequent revisions continued to show significant decreases in LSSS scores: a decrease of 9.52 points after the first revision (LSSS2), 10.65 points after the second revision (LSSS3), and 11.24 points after the third revision (LSSS4), all with p-values less than 0.001. These results indicate that VNS surgery and subsequent revisions are associated with progressively reduced seizure severity in the adult population. The model also showed that age had no significant effect on LSSS scores (p = 0.465), emphasizing that improvements in seizure severity were primarily related to surgical interventions. LSSS: Liverpool Seizure Severity Scale; CI: confidence interval

Predictors	LSSS 2.0 estimations	CI	p-value
Reference LSSS (LSSS0)	47.32	(40.15 to 54.49)	<0.001
Installation (LSSS1)	-8.38	(-11.39 to -5.37)	<0.001
1st revision (LSSS2)	-9.52	(-12.97 to -6.06)	<0.001
2nd revision (LSSS3)	-10.65	(-15.18 to -6.13)	<0.001
3rd revision (LSSS4)	-11.24	(-15.99 to -6.48)	<0.001
Age	0.05	(-0.08 to 0.17)	0.465

Evaluation of changes in seizure severity in the pediatric population

**Figure 6:**
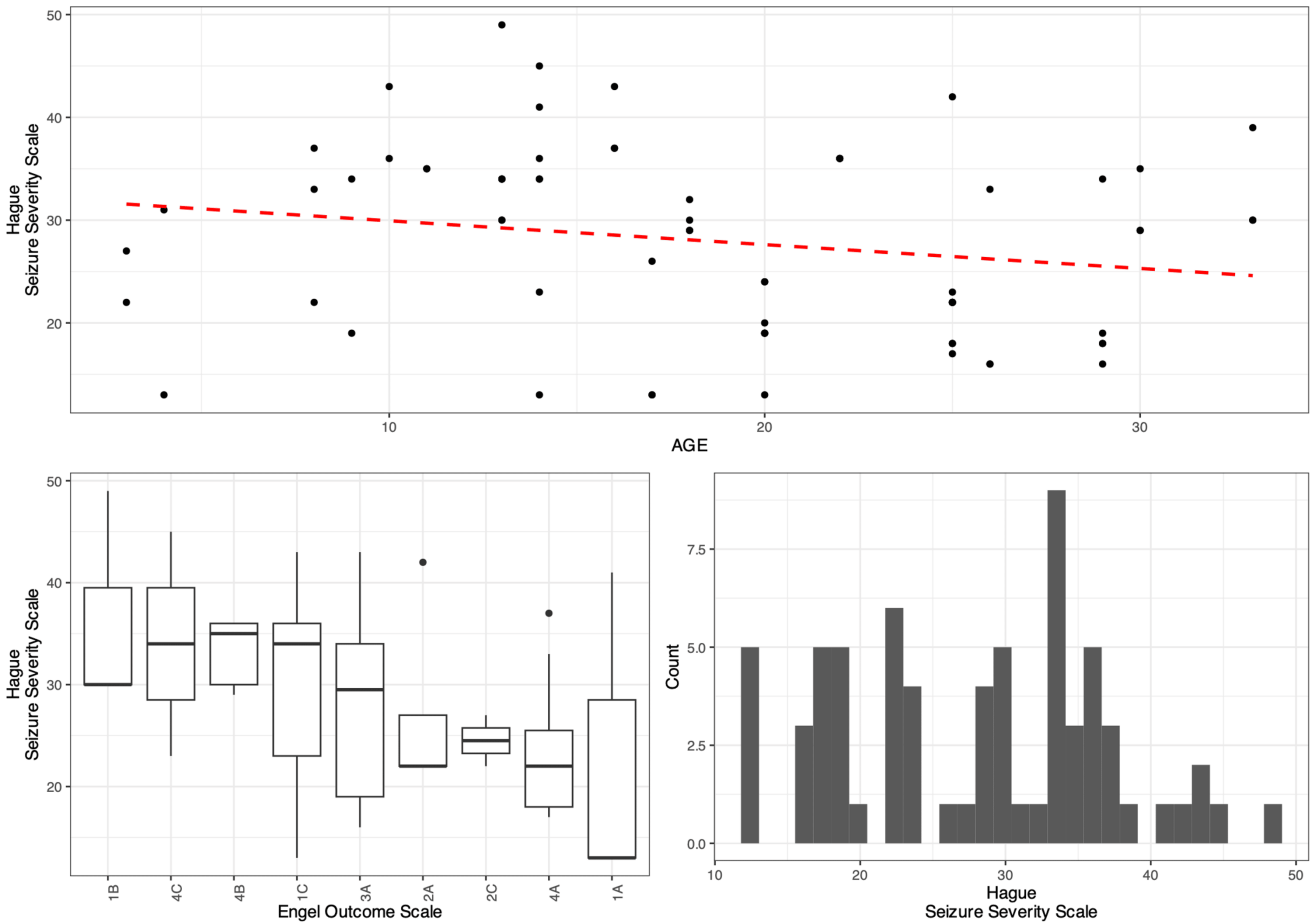
Diagrams illustrating the relationship between Engel Outcome Scale and Hague Seizure Severity Scale in the pediatric population The figure demonstrates that improved Engel Outcome Scale (EOS) outcomes are associated with lower Hague Seizure Severity Scale (HASS) scores, reflecting reduced seizure severity in pediatric and cognitively impaired patients. The mixed-effects linear regression model revealed that HASS scores decreased significantly after each surgery, with an average reduction of 5 points after the initial installation (p < 0.001) and further reductions following each revision, highlighting the effectiveness of vagus nerve stimulation (VNS) in reducing seizure severity in the pediatric and cognitively impaired population.

The scatter plot in Figure 6 indicates that the HSSS decreases modestly with age, while the boxplots show that the Engel Scale exposes substantial patient differences in HSSS with respect to their median patient HSSS. Finally, the histogram shows that HSSS is not normally distributed and rather uniform before midlife, giving a positive skew, with a median of 29.375 (19.35-34.75).

A mixed-effect linear regression model which contained the EOS scores as a random effect was fit to the data in a step-wise-step up procedure. Due to the presence of outliers in the data, weights were included in the model which led to a signiﬁcantly improved model fit compared to an un-weight model (χ^2^ (2): 39.46, p < 0.0001).

The final minimal adequate model performed signiﬁcantly better than an intercept-only base-line model (χ^2^ (1): 46.82 < 0.0001) and showed that HSSS decreases significantly and substantially after surgery (HSSS0 ~ 31.36 vs. HSSS1 ~ 26.36).

With each revision, a three-point decrease is evident (p < 0.001, marginal R^2^ = 0.145, conditional R^2^ = 0.599). Age was revealed to be worthless in improving the model. Moreover, a patient's grade on Engel's scale affects how the HSSS, specifically the standard deviation between a patient's HSSS and another accounting for one's Engel and revision is 5.46 points. A more detailed progression of the model can be visualized in Table 4.

**Table 4:** Mixed effects multiple linear regression model for the pediatric population using the Hague Seizure Severity Scale The table presents the estimated effects of vagus nerve stimulation surgeries and revisions on HSSS scores for pediatric patients and those with impaired cognitive and communicative abilities. The baseline HSSS score (HSSS0) before any intervention was 31.36. After the initial VNS installation (HSSS1), there was a significant reduction of 5 points (p < 0.001). Further decreases in HSSS scores were observed with subsequent revisions: an 8.07-point reduction after the first revision (HSSS2), an 11.23-point reduction after the second revision (HSSS3), and a 13.8-point reduction after the third revision (HSSS4). These reductions were all statistically significant, with p-values less than 0.05. These results indicate that VNS surgery and subsequent revisions are associated with progressively reduced seizure severity in the pediatric and cognitively impaired population, demonstrating the effectiveness of VNS therapy across multiple interventions. HSSS: Hague Seizure Severity Scale (representing corresponding score); CI: confidence interval

Predictors	HSSS estimations	CI	p-value
Reference HSSS (HSSS0)	31.36	(25.72 to 36.99)	<0.001
Installation (HSSS1)	-5	(-5.00 to -5.00)	<0.001
1st revision (HSSS2)	-8.07	(-12.04 to -4.10)	<0.001
2nd revision (HSSS3)	-11.23	(-16.77 to -5.69)	<0.001
3rd revision (HSSS4)	-13.8	(-25.13 to -2.48)	0.022

Changes in seizure frequency

The data was categorized into 3 levels: 1 (several times per month), 2 (less than once per month), and 3 (no tremor in the year preceding/following the surgery). Fisher's exact test for count data was used due to the presence of counts <5.

Test results reveal a p-value ≤ 0.001, which indicates that the average number of seizures is different before and after surgery. Specifically, having several seizures a month appeared to be significantly lower after surgery (p-value = 0.5343 when not counting 0 surgeries in, otherwise post-hoc p-values < 0.01695).

The distribution of frequencies can be visualized in Table 5.

**Table 5:** Distribution of frequency scores before and after vagus nerve stimulation surgery The table presents the number of patients categorized by seizure frequency before and after undergoing VNS therapy. Seizure frequency was classified into three categories: 1 (several times per month), 2 (less than once per month), and 3 (no tremors in the year preceding/following the intervention). Before VNS surgery, 46 patients experienced seizures several times per month, three patients had seizures less than once per month, and no patients were seizure-free. After VNS surgery, the number of patients experiencing seizures several times per month decreased to 27, while those experiencing seizures less than once per month increased to 15. Notably, seven patients reported being seizure-free in the year following surgery. These results demonstrate the effectiveness of VNS therapy in significantly reducing seizure frequency among patients with multifocal refractory epilepsy. VNS: vagus nerve stimulation

Frequency score	Count before VNS	Count after VNS
1	46	27
2	3	15
3	0	7

Changes in QoL and mood

**Figure 7:**
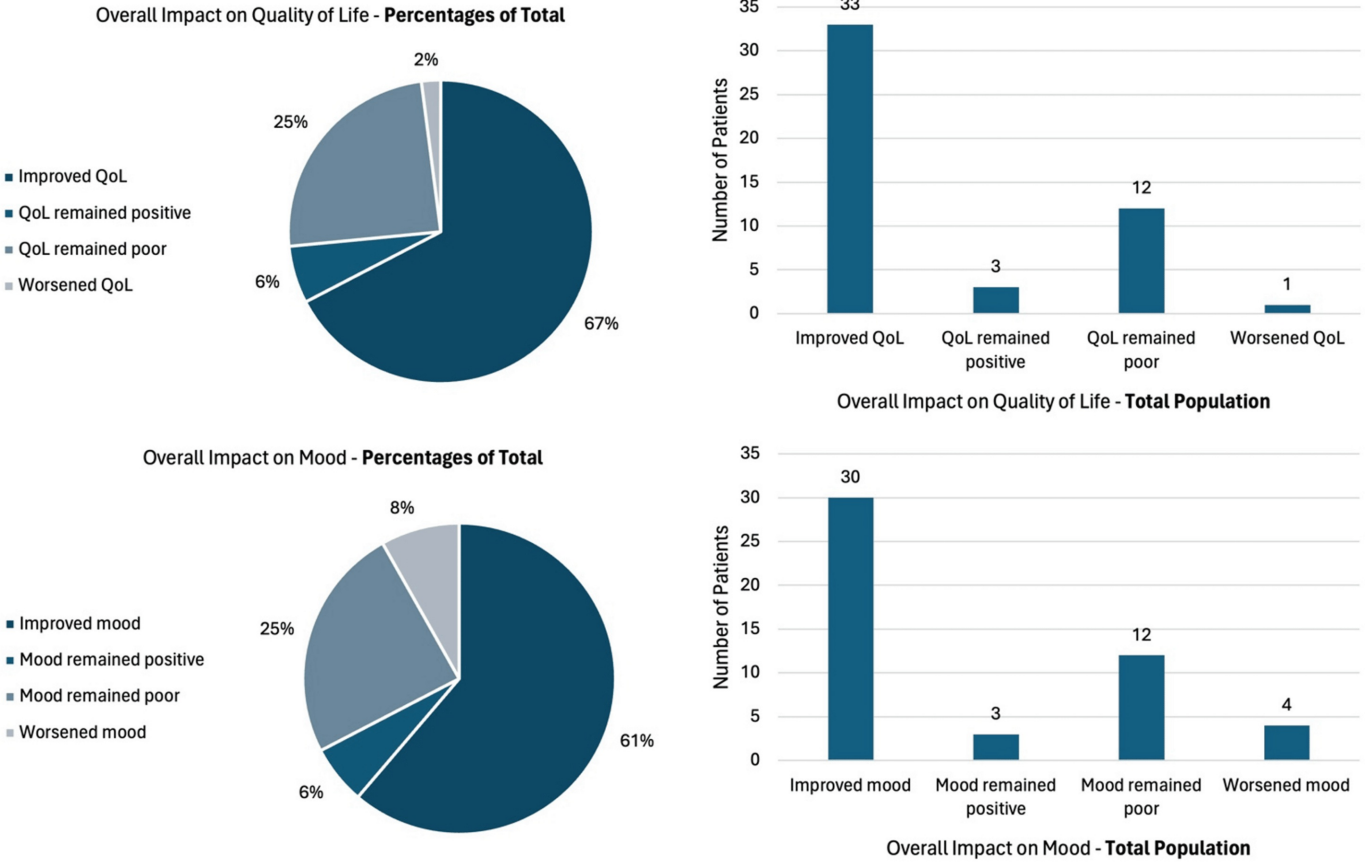
Diagrams illustrating the overall impact of vagus nerve stimulation on quality of life and mood The figure shows that the majority of patients reported improvements in quality of life and mood after VNS surgery. Specifically, 67% of patients indicated improved quality of life, and 61% reported enhanced mood, highlighting the broader benefits of VNS therapy beyond seizure control. QoL: quality of life; VNS: vagus nerve stimulation

As previously described, changes in QoL and mood were reported based on two questions with four answers each. This scale already has a subjective temporal component for changes in these parameters. The proportional results are observed in Figure 7.

It is noted that the vast majority of patients presented a positive result. However, it will be necessary to consider the subjective nature of this result and consider other scales or methods of evaluation of these two parameters in future studies, specific to this context.

Changes in the number of hospitalizations

The number of hospitalizations considered for this study included hospitalizations directly related to epilepsy; hospitalizations for other causes were not included.

The data was categorized into seven levels: 0, 1, 2, 3, 4 and 5. The Fisher's exact test for categorical data was used due to the presence of counts less than 5.

The test results reveal a p-value < 0.001, indicating that the number of hospitalizations is different before and after surgery. More specifically, having no crises appeared significantly higher after surgery (p-value = 0.6289, excluding surgeries with 0). The distribution of numbers can be observed in Table 6.

**Table 6:** Distribution of hospitalizations before and after vagus nerve stimulation surgery The table shows a significant increase in the number of patients with no hospitalizations (from 21 to 42) after VNS and a decrease in those with more frequent hospitalizations. This indicates that VNS therapy effectively reduces hospitalizations in patients with multifocal refractory epilepsy. VNS: vagus nerve stimulation

Number of hospitalizations	Count before VNS	Count after VNS
>5	4	1
0	21	42
1	7	1
2	3	3
3	6	1
4	2	0
5	6	1

Changes in the number of medications

The data turned out to be normally distributed and to have equal variance. Since the data came from the same population at different times, the paired Student's t-test was used.

The test revealed a p-value of (p = 0.185), indicating that the number of medications before and after is not statistically different.

## Discussion

The main outcome measures of this study were seizure severity scale variation before and after surgery, seizure frequency reduction (or increase), QoL, mood variations, outcome scales, and adverse events. The scales used in our study were all validated in English. The LSSS is classically used in the context of prospective VNS studies [15-17] and it has also been used in the retrospective format [18,19]. The seizure severity scales have been validated and updated in English [9] and other languages [10,20]. The HSSS is a less commonly used severity scale. HSSS evaluated the severity of seizures in pediatric patients and patients who are not able to answer questions by addressing the questions to their main caregiver [11,12]. To our knowledge, the HSSS has never been used to evaluate VNS in children nor has it been used in a retrospective setting. However, it has been used in other studies to prospectively evaluate various other treatment options for pediatric epilepsy [21-24]. The EOS is a widely used scale to evaluate the effectiveness of epilepsy surgery especially temporal lobe resections. It first emerged in a study published in 1993 by Engel [25]. This scale has not been used to assess VNS in a prospective study until the writing of this article. However, it has been used in retrospective studies and has shown validity [26,27]. Although this is the first time HSSS is used retrospectively in a VNS study, its correlation with EOS as shown in the results retroactively justifies its use as the EOS is already validated in this context. Thus, the data collected in this study according to those three scales are interpretable and a reliable assessment of the surgical outcome of the VNS surgeries conducted.

Refractory epilepsy is defined by the International League Against Epilepsy (ILAE) as epilepsy that hasn’t been controlled under two well-tolerated and properly administered AED [8]. The most common causes of refractory epilepsy are usually surgically treatable causes such as brain tumors or focal epilepsy as these causes are usually harder to treat than idiopathic epilepsy [28]. However, VNS is not indicated in all refractory epilepsy cases, it is only recommended in refractory epilepsy patients who cannot undergo intracranial surgical treatment (either because of a lack of indication or because of patient refusal) or in cases where surgical treatment failed to control seizures [29]. VNS has shown low variability in efficacy between people with different underlying etiology of epilepsy and thus is indicated regardless of the cause of the epileptic disorder [30]. As mentioned previously, the patients included have different etiologies and most of them have idiopathic epilepsy. All of these patients met the required criteria for treatment with VNS and no difference in outcome between different etiologies was observed.

VNS is also known to be a simple procedure with very few major side effects [31]. Similarly in this study, most side effects were minor and did not generate a significant concern to the treating physician or the patient. An interesting observation was the fact that people suffering from a side effect in the first surgery were more likely to suffer from the same side effect permanently, even after multiple revisions, especially in the case of people experiencing dysphonia. It is unclear whether this phenomenon is in harmony with the literature; it is however important to note that dysphonia is the most common side effect observed three months after surgery (62%) and only partly decreases in frequency after 12 months of follow-up (55%). This adverse event nevertheless decreases significantly in frequency at the five-year follow-up (18.7%) [32]. The permanence of side effects observed in our study could be caused by the recall bias inherently present by the nature of the study design. Intuitively, patients would preferably report side effects that are lasting while forgetting about those that disappeared a long time ago, especially considering that the side effects are mostly minor as mentioned previously, and thus do not constitute a memorable experience for the patient.

No significant variation in AED consumption was observed in the case of our patients. This is standard after VNS surgeries, most patients continue to require medical treatment after the procedure with only a few patients experiencing a reduction in drugs needed to control epilepsy [33]. An observed improvement with time after surgery in our patients is worth mentioning, meaning the patients had improved outcomes after surgery. However, most patients only had a battery change in revision surgeries thus no major modification was done to the apparatus. This could be attributed to a well-described phenomenon after VNS surgery in which patients seem to have increasing seizure control and responsiveness to stimulation with time [34]. And naturally, since the only recorded data in this study was after each surgery, this phenomenon revealed itself in this manner. Additionally, a significant reduction in the frequency of seizures was observed, as well as an improvement of seizure severity (whether assessed by LSSS or HSSS) and only a quarter of patients experienced no worthwhile improvement according to the EOS. This highlights both the viability of VNS as a treatment of refractory epilepsy and its effect on most if not all the negative aspects of the epileptic syndromes (seizure frequency, seizure severity, QoL, etc.). The number of hospitalization and ER visits due to epilepsy-related events was also significantly decreased further supporting this argument.

The main limitation of this study was the recall bias. This being a retrospective study based on a questionnaire makes this limitation the most prominent. However, the study design was built to accommodate this bias in a suitable manner without it significantly influencing the results. To start, most of the questions asked were about memorable experiences that patients or their main caregivers could easily remember. Furthermore, some measures like the frequency of seizures were divided into broad categories so patients should not have to answer with a specific number but rather had to recall, for example, if the seizures were happening more than once a month or less than once a month which was made easy for most patients. Moreover, the data collection included a rigorous analysis of the patient’s medical records from different resources which also narrows the amplitude of effect this bias could have on the final results. Finally, a considerable portion of patients were evaluated directly after surgery thus generating reliable, real-time data. Other limitations include the subjective nature of some of the outcome measures like questions asked about mood and the QoL before and after the surgery. However, this does not constitute a real problem since a patient-centered analysis is preferable and since other measures are objective and the results of subjective and objective parameters were in harmony. A final limitation worth mentioning is the lack of documentation for some side effects like sleep apnea, this was overcome by asking for all sleep disturbances without needing a diagnosis of obstructive sleep apnea syndrome.

This study is the first that uses the HASS, LSSS, and EOS at the same time to evaluate seizure severity reduction after VNS while also evaluating QoL, mood improvement, and seizure ⁠⁠frequency reduction. The multimodal assessment of the outcome of VNS, and the results significantly displayed, emphasize the importance of studies like this one that incorporate most of the aspects of the life of an epileptic patient. This retrospective study design should inspire prospective studies to take a holistic approach in the evaluation of patients before and after VNS surgery.

## Conclusions

In conclusion, this study presents a quantifiable model for evaluating VNS outcomes, demonstrating its potential utility in managing multifocal refractory epilepsy. While our findings suggest significant improvements in seizure severity and QoL metrics, further research with larger cohorts and diverse subgroups is necessary to confirm these results and refine the model. This study provides a foundation for future investigations into the efficacy of VNS and underscores the importance of comprehensive outcome assessment in epilepsy treatment.
